# Lesional Intractable Epileptic Spasms in Children: Electroclinical Localization and Postoperative Outcomes

**DOI:** 10.3389/fneur.2022.922778

**Published:** 2022-07-22

**Authors:** Shuang Wang, Chang Liu, Hongwei Zhang, Qingzhu Liu, Taoyun Ji, Ying Zhu, Yan Fan, Hao Yu, Guojing Yu, Wen Wang, Dongming Wang, Lixin Cai, Xiaoyan Liu

**Affiliations:** ^1^Pediatric Epilepsy Center, Peking University First Hospital, Beijing, China; ^2^Department of Pediatrics, Peking University First Hospital, Beijing, China; ^3^Department of Neurology, Qilu Children's Hospital of Shandong University, Shandong, China; ^4^Department of Radiology, Peking University First Hospital, Beijing, China; ^5^Department of Nuclear Medicine, Peking University First Hospital, Beijing, China

**Keywords:** epileptic spasm, semiology, EEG, imaging, epilepsy surgery

## Abstract

To analyze the influence of seizure semiology, electroencephalography (EEG) features and magnetic resonance imaging (MRI) change on epileptogenic zone localization and surgical prognosis in children with epileptic spasm (ES) were assessed. Data from 127 patients with medically intractable epilepsy with ES who underwent surgical treatment were retrospectively analyzed. ES semiology was classified as non-lateralized, bilateral asymmetric, and focal. Interictal epileptiform discharges were divided into diffusive or multifocal, unilateral, and focal. MRI results showed visible local lesions for all patients, while the anatomo-electrical-clinical value of localization of the epileptogenic zone was dependent on the surgical outcome. During preoperative video EEG monitoring, among all 127 cases, 53 cases (41.7%) had ES only, 46 (36.2%) had ES and focal seizures, 17 (13.4%) had ES and generalized seizures, and 11 (8.7%) had ES with focal and generalized seizures. Notably, 35 (27.6%) and 92 cases (72.4%) showed simple and complex ES, respectively. Interictal EEG showed that 22 cases (17.3%) had bilateral multifocal discharges or hypsarrhythmia, 25 (19.7%) had unilateral dominant discharges, and 80 (63.0%) had definite focal or regional discharges. Ictal discharges were generalized/bilateral in 71 cases (55.9%) and definite/lateralized in 56 cases (44.1%). Surgically resected lesions were in the hemisphere (28.3%), frontal lobe (24.4%), temporal lobe (16.5%), temporo-parieto-occipital region (14.2%), and posterior cortex region (8.7%). Seizure-free rates at 1 and 4 years postoperatively were 81.8 and 72.7%, respectively. There was no significant difference between electroclinical characteristics of ES and seizure-free rate. Surgical treatment showed good outcomes in most patients in this cohort. Semiology and ictal EEG change of ES had no effect on localization, while focal or lateralized epileptiform discharges of interictal EEG may affect lateralization and localization. Complete resection of epileptogenic lesions identified *via* MRI was the only factor associated with a positive surgical outcome.

## Introduction

Epileptic spasm (ES), a common seizure type of medically intractable pediatric epilepsy, is more common in young children. Video electroencephalogram (VEEG) has revealed that in addition to typical ES and hypsarrhythmia, several ESs are characterized by bilateral asymmetry or focal symptoms, often with focal epileptiform discharges in interictal electroencephalography (EEG). A considerable portion of patients with ES show focal epileptogenic lesions in brain magnetic resonance imaging (MRI), and surgical treatment has shown to be effective. Therefore, according to the 2017 International League Against Epilepsy Seizure Type Classification, ES was divided into three types for the first time, namely, focal, generalized, or unknown onset (1, 2). However, systematic research on the importance of localization on ES semiology and of EEG characteristics in the presurgical evaluation and their impact on epilepsy surgery outcomes are limited, and the validation of the previous study's results was limited due to the small available sample size. In this study, we retrospectively studied children with medically intractable epilepsy who had ES and received surgical resection or disconnection (hereinafter referred to as surgical treatment) in a large cohort (127 patients), to analyze the influencing factors of ES in the epileptogenic zone (EZ) localization and surgical prognosis, especially the localization value on ES semiology and EEG findings.

## Methods

We reviewed patients who received surgical treatment at the pediatric epilepsy center of Peking University First Hospital from May 2014 to December 2018. This study was approved by the Institutional Review Board of the Ethics Committee of Peking University First Hospital. Presurgical evaluations included detailed historical and etiological investigations (some patients were tested by whole-exome sequencing and copy number variant), ictal and interictal VEEG monitoring, high resolution 3.0T MRI epilepsy protocol, fluorodeoxyglucose positron emission tomography (FDG-PET) and co-registration of PET and MRI, and assessment of Griffiths development and Peabody motor development scales. ES was defined as a short clustered, abnormal contraction of the trunk, bilateral limbs, or focal muscles (eyes, mouth angle, or unilateral limb), with each cluster including ≥ 3 spasms; the duration of electromyogram (EMG) in each spasm was defined as 0.5–2 s (longer for the first spasms cluster) (3). Etiology was determined *via* a comprehensive analysis of past medical history, MRI findings, and postoperative pathology.

The inclusion criteria were as follows: (1) medically intractable epilepsy under 18 years old; (2) clustered ES recorded in the last VEEG before surgery, with or without other types of focal or generalized seizures; (3) focal or hemispheric structural abnormality on MRI, accompanied by hypometabolism in the corresponding PET cortical area (a few were hypermetabolism); (4) all patients underwent resective surgery (excluding the vagus nerve stimulation (VNS) or corpus callosotomy); and (5) postoperative follow-up ≥ 12 months (the second postoperative follow-up time should be taken as the standard for patients who underwent two surgeries). Conversely, exclusion criteria were as follows: (1) not being a surgical candidate, defined as definite or high suspicion of unsuitable causes for surgical treatment, such as bilateral or extensive brain structural abnormalities, or detection of pathogenic gene variants unsuitable for surgical treatment; (2) negative MRI findings; and (3) FDG-PET showing bilateral extensive or multiple focal hypometabolic regions (except tuberous sclerosis complex).

### Imaging

All imaging modalities, including MRI and co-registration of MRI and PET, were evaluated by neuroradiologists, pediatric epileptologists, surgeons, and neurophysiologists during the multidisciplinary presurgical evaluation. No seizures were detected during PET scans for all patients. For areas of obvious focal hypermetabolism, we considered that such abnormal hypermetabolism was related to continuous epileptiform discharges according to recent VEEG. A focal epileptogenic structural abnormality detected on MRI is the basic condition for surgical treatment. Surgical treatment is not considered for ES only with a focal metabolic abnormality on PET but still negative on MRI after reevaluation, regardless of focal epileptiform discharges on EEG.

### Electroclinical Classification of ES

The ES semiology was classified according to presurgical VEEG and divided into three groups as follows: (1) symmetric ES, including symmetrical, bilateral limb ES, or only nodding and axial spasm without limb movement; (2) asymmetric ES, with obvious asymmetrical, bilateral, or unilateral limb ES, and/or evolving into symmetrical ES; and (3) focal ES (confined to the unilateral upper or lower limb, eyelid, eyeball, or perioral region), or ES accompanied by transient stereotyped hypermotor-like movement, and focal ES evolving into symmetric or asymmetric ES during one clustered seizure.

The ES can be further classified as simple when all ESs are from the same group as described above, or complex when more than one of the above features is seen. ES may be the only seizure type detected by VEEG preoperatively, or other types of focal or generalized seizures may be recorded in the same VEEG monitoring course or in the same episode. Within the same episode, other seizure types mostly appear before the ES.

Interictal epileptiform discharges were divided into three groups as follows: (1) generalized or multifocal discharges, with no definite unilateral or focal characteristics during the whole recording; (2) constant lateralized discharges, with or without contralateral discharges; and (3) constant focal or regional discharges, with or without generalized or multifocal discharges. Ictal epileptiform discharges were further divided into generalized and lateralized.

### Surgery and Outcome

Surgical resection was guided by MRI structural abnormalities, combined with co-registration of PET and MRI, semiology, presurgical VEEG, and intraoperative electrocorticography (EcoG) monitoring. The scope of resection was divided by lobes, but the central region (precentral and postcentral gyrus) was regarded as an independent cortical area. Surgical outcomes were classified according to the Engel's classification, and Engel Ia was looked at as seizure-free.

### Statistical Analysis

SPSS 20.0 (IBM Corp., Armonk, NY, USA) was used to analyze the relationship between ES semiology, ictal-interictal EEG, location and scope of surgical resection, and postoperative seizure outcome. Data distribution normality was determined; non-normally distributed data are presented as medians (interquartile intervals), and the non-parametric Mann-Whitney *U* rank-sum test was used for comparison between groups; discrete data are presented as frequencies and percentages. The chi-square and Fisher exact probability tests were used for between-group comparisons. *P* < 0.05 was considered statistically significant.

## Results

Overall, 567 children with medically intractable epilepsy underwent resective or disconnective surgery at our epilepsy center from May 2014 to December 2018. In addition, 127 individuals (78 males, 49 females, 22.4%) had ESs detected by presurgical VEEG monitoring. The seizure onset age ranged from 2 h after birth to 100 months (median age 7 months); 117 children (92.1%) were ≤3 years old. The age at surgery ranged from 7 to 205 months (median age 35 months); 65 children (51.2%) were ≤3 years old at surgery. The seizure duration was 3–200 months (median 25 months).

All patients had focal structural epileptogenic abnormalities, including focal cortical dysplasia, malformation of cortical development (MCD), or long-term epilepsy-associated brain tumors (LEATs) in the presurgical MRIs in 97 cases (76.4%), while early acquired brain structural injury was seen in 30 cases (23.6%). Presurgical PET imaging was performed in 99 cases. The co-registration of PET and MRI showed 95 cases (96.0%) of abnormal focal hypometabolism and 4 cases (4.0%) of abnormal focal hypermetabolism. Furthermore, the abnormal metabolic area was consistent with or larger than the structural lesion.

In the 127 presurgical VEEG studies, 53 (41.7%), 46 (36.2%), 17 (13.4%), and 11 cases (8.7%) had ES only; ES and focal seizures; ES and generalized seizures (including myoclonus, atypical absence, atonic, or tonic seizures); and ES, focal seizures, and generalized seizures, respectively. There were 35 cases (27.6%) of simple ES and 92 (72.4%) of complex ES. Except for one case in which only eyelid ES was seen, other focal eyeball, eyelid, or mouth angle ES were accompanied by symmetric ES or asymmetric ES. Of the 127 included children, 64 (50.4%) had symmetric ES, while 48 (37.8%) had asymmetric ES; 79 cases (62.2%) had focal ES.

Interictal EEG showed that 22 cases (17.3%) had multifocal epileptiform discharge or hypsarrhythmia, 25 (19.7%) had lateralized discharge, and 80 (63.0%) had a definite focal or regional discharge. Ictal EEG of ES showed that 71 cases (55.9%) had generalized ictal discharge, and 56 (44.1%) had definite lateralized ictal discharge.

**Figure 1:**
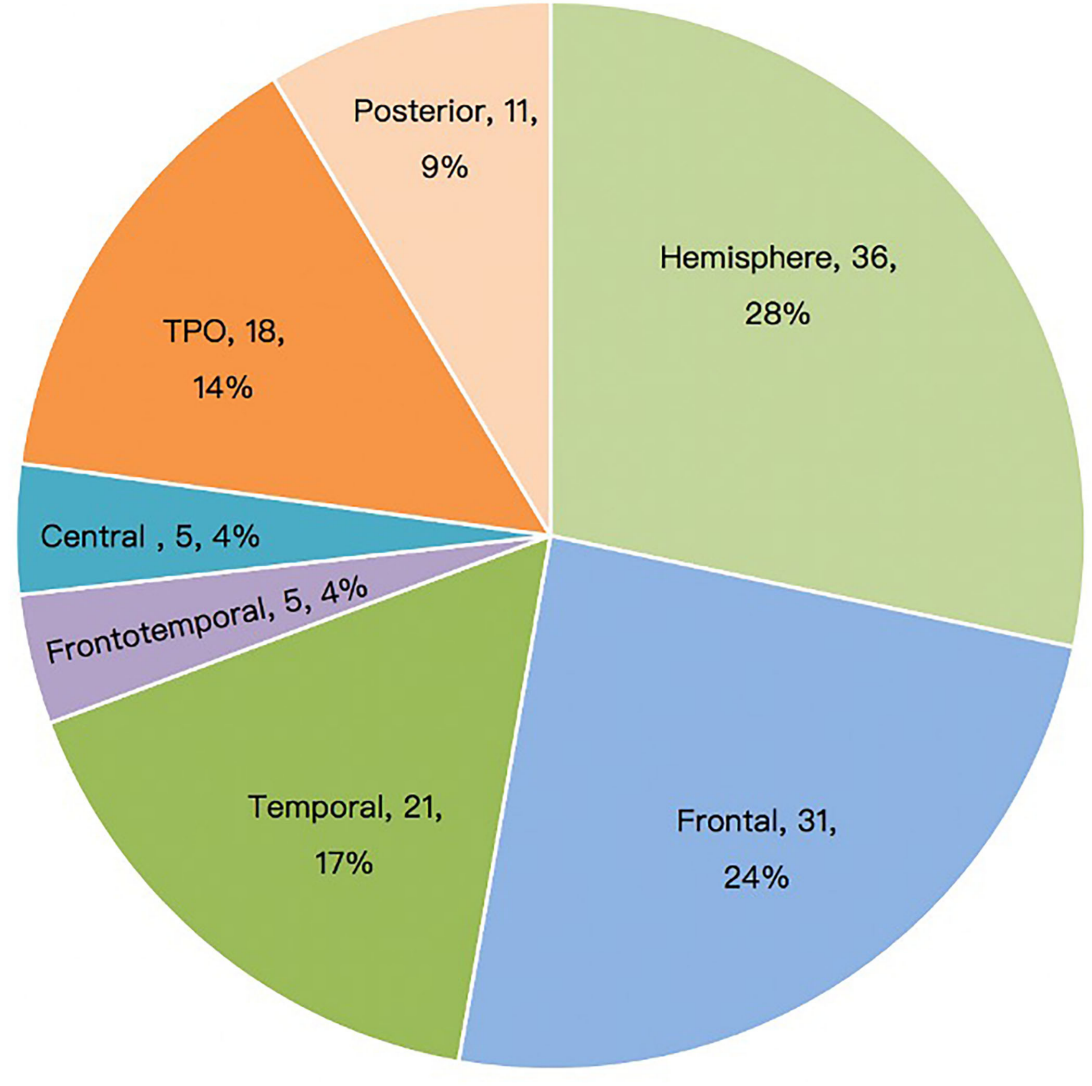
Surgical sites of patients with epileptic spasm (*n* = 127). TPO, temporo-parieto-occipital.

Among the 127 patients with ES, intra-lobar focal EZ resection was performed in 29 cases (22.8%); unilobar resection or disconnection in 41 (32.3%); multilobar resections and disconnections in 21 (16.5%); and hemispherotomy in 36 (28.3%). Some insular structural parts were involved in frontal or temporal lobe (including peripheral lateral fissure) surgery; however, no single insular resections were performed. The distribution of resection or disconnection location is summarized in Figure 1.

**Figure 2:**
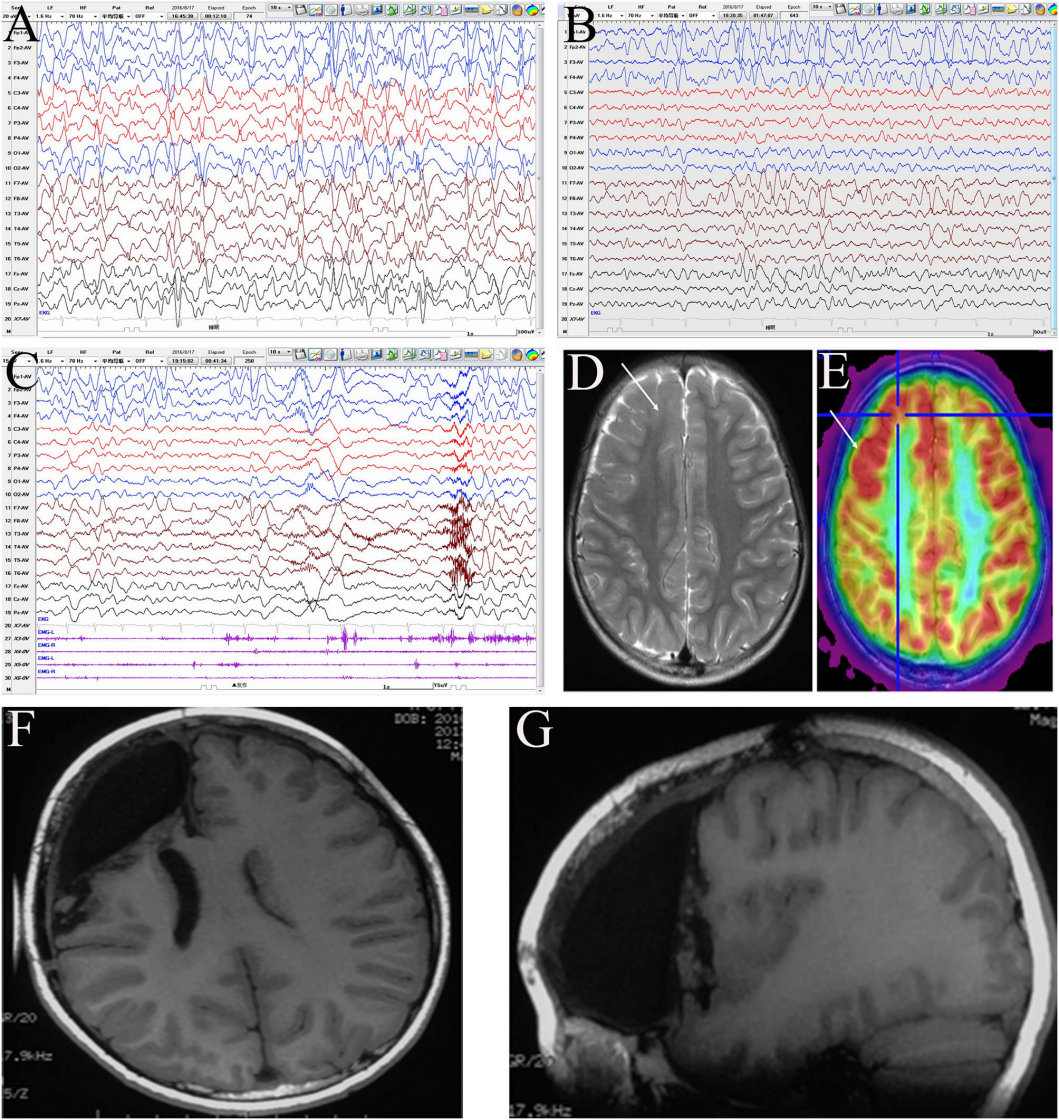
Evaluation of a male patient, 5 years old with onset age at 1 year and 6 months and clustered asymmetric epileptic spasm, which was obvious in the left upper limb. The patient had been prescribed several anti-seizure medicines, including adrenocorticotropic hormone, valproic acid, topiramate, and levetiracetam, with no success. The patient had mental and motor developmental delay and no obvious hemiplegia. Interictal electroencephalogram (EEG): hypsarrhythmia **(A)**, with intermittent right frontal regional continuous discharge (FP2, F4, and F8) **(B)**; ictal EEG: fast rhythm dominated on the right hemisphere **(C)**. MRI: focal cortical dysplasia (FCD) in the right frontal lobe **(D)**, with boundary-clear focal hypermetabolism on positron emission tomography (PET) **(E)**. According to the presurgery evaluation and intraoperative electrocorticography, the resection scope included the right prefrontal lobe, anterior cingulate cortex, frontal central operculum, and anterior insula **(F,G)**. Pathology: a few dysmorphic neurons (FCD IIA) in the ACC and malformation of cortical development in other areas. The patient has been seizure-free for 3 years and shows mild mental and motor progression.

A representative EEG and preoperative and postoperative imaging findings of a case of right frontal lobe lesion with ES are presented in Figure 2.

**Figure 3:**
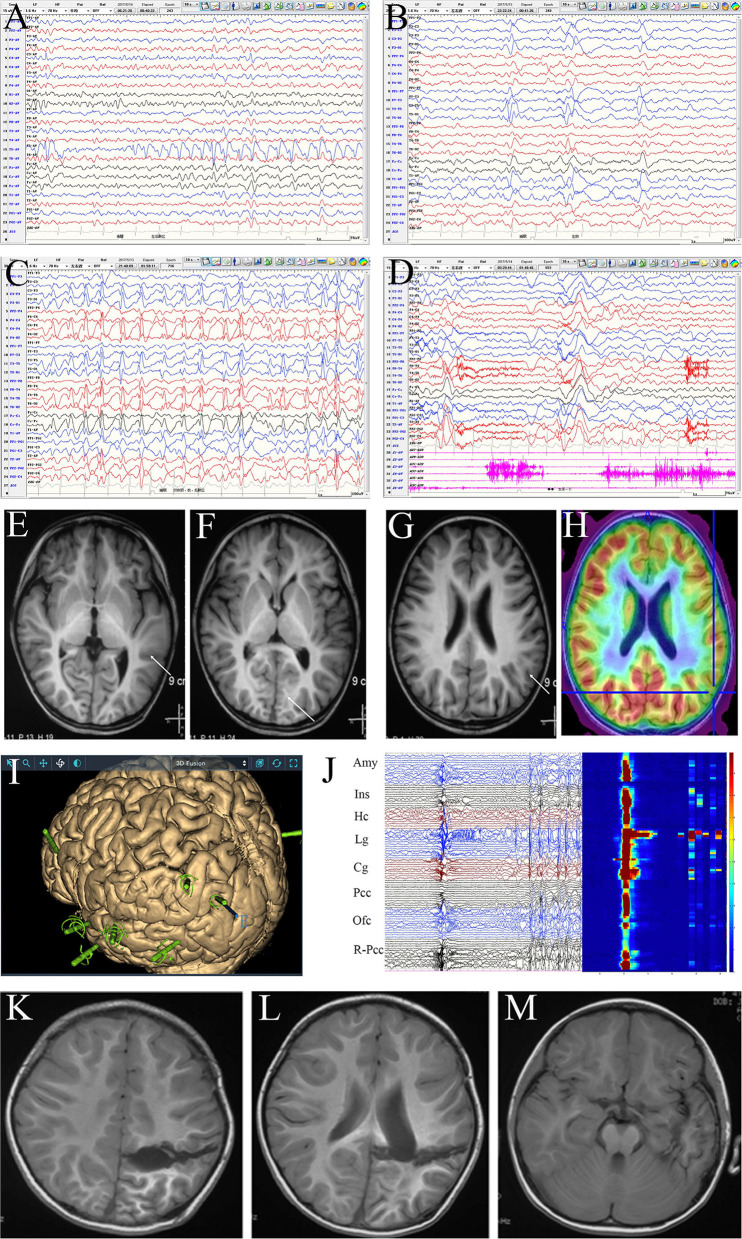
Evaluation of a female patient, 4 years and 4 months old, with onset age at 3 years and 3 months and clustered or isolated symmetrical epileptic spasm (ES) as well as frequent daily seizures. The patient had been prescribed several anti-seizure medicines, including adrenocorticotropic hormone, vigabatrin, valproic acid, topiramate, and levetiracetam, with no success, and had mild developmental delay; she was accepted for presurgical evaluation at 4 years old. Scalp interictal EEG showed frequent discharges dominating in the left temporo-parieto-occipital area, which spread diffusively on both hemispheres **(A–C)** and was accompanied by atypical absence seizures and ES **(D)**. MRI: focal cortical dysplasia in the left neocortical temporal lobe. No definite structural abnormality was seen in the left parietal-occipital region **(E–G)**; PET-MRI: mild hypometabolism of the left inferior parietal lobe and parietal operculum **(H)**. Given the discordance of epileptic zone localization information, stereoelectroencephalography monitoring was performed. The electrodes covered the left parietal, occipital, temporal, insular, and posterior cingulate cortices (Pcc), as well as the orbital frontal cortex (Ofc) and right parieto-occipital junction areas **(I)**. Interictal and ictal EEG: most areas showed synchronous discharges (including contralateral) except for the hippocampus (Hc), amygdala (Amy), insula (Ins), and Pcc. The discharges in the lingual gyrus (Lg) and cuneus gyrus (Cg) appeared 20–30 ms in advance in each ES, and an after-discharge was seen in the lingual gyrus. The time-frequency diagram showed that the highest high-frequency energy was found in the anterior lingual and inferior Cg **(J)**. Disconnection of temporo-parieto-occipital lobes was performed **(K,L)**, while the Amy and Hc were preserved in the mesial temporal lobe **(M)**. Pathology: malformation of cortical development. At the 3-year follow-up, the patient was seizure-free and showed mental and motor development progression.

Notably, 7 patients, all older than 3 years, underwent intracranial EEG monitoring, including four in which subdural and deep electrodes were used before 2017 and three in whom stereoelectroencephalography (SEEG) was performed after 2017. Such invasive studies were performed when the lesions were located in the motor or language eloquent cortex, or in the insular or mesial lateral regions, or when the MRI, PET, and interictal EEG results were incongruent. Intracranial EEG showed that the ictal high-frequency rhythm of ES propagated rapidly to most or all electrodes and was most obvious in some contacts inside the lesion. This rhythmic activity appeared approximately 100 ms ahead of time in the focal region, or had a short post-ES after-discharge, while this focal initiative feature usually only appeared in some of the ESs in the same patient. However, in ictal intracranial EEG, if the brain area covered by electrodes is clearly uninvolved, they must be preserved during surgery (Figure 3).

Of the four patients who had both subdural and deep electrode implantations, two became seizure-free, while the other two had ongoing seizures. Of the three patients who underwent SEEG, one was seizure-free and the other two had ongoing seizures. Of the 127 patients with ES, 9 (7.1%) underwent a second expanded resection or hemispherotomy due to very frequent seizures. Among patients who underwent second resective surgery, six became seizure-free and three had ongoing seizures.

**Figure 4:**
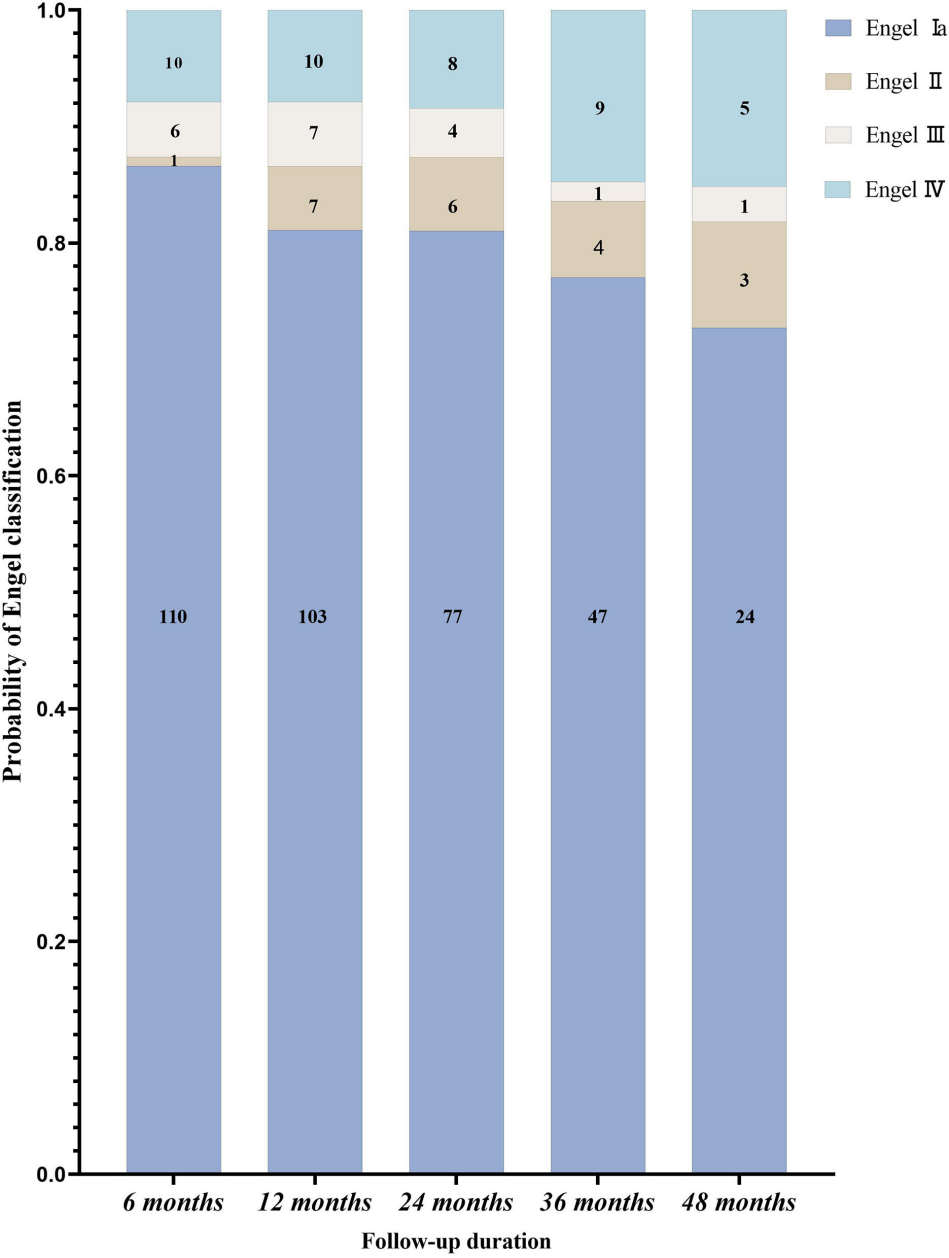
The postoperative follow-up of patients with epileptic spasms according to the Engel classification (*n* = 127).

The follow-up time was 12–48 months. Postoperative outcomes are shown in Figure 4. Of the 127 patients, the seizure-free rate was 81.1% (103/127) at 1 year and 72.7% (24/33) at 4 years. The overall seizure-free rate was 79.5% (101/127).

There were no significant differences in seizure-free rates across different surgical sites. The relationship between ES semiology and surgical sites in the 101 seizure-free children after surgery is shown in Table 1. Bilateral asymmetric ES (33 cases, 32.7%) and single-limb ES (18 cases, 17.8%) all occurred in the contralateral surgical site limb, especially the upper limb. All five patients with unilateral lower limb ES had a recurrence after surgery. The surgical sites were the hemisphere, TPO area, and temporal lobe in one patient each, while there were two patients who underwent frontal lobe surgery. Focal ES with eyeball, eyelid, or mouth angle involvement was seen in 42 cases (41.6%), corresponding to surgical sites in multiple cortical areas. One patient with a cluster of ESs with features similar to hypermotor seizures had a frontal lobe lesion. The data scattering of ES features and surgical sites did not allow for statistical analysis. Among 26 patients with postoperative recurrence, five (19.2%) had simple ES and 21 (80.8%) had complex ES.

**Table 1:** Relationship between ES semiology and surgical sites in seizure-free patients after surgery (*N* = 101).

**Surgical sites** **Number** **(seizure-free rate %)**	**Symmetric ES**		**Asymmetric ES**	**Focal ES**					
	**Bilateral** **symmetry**	**Nod** **only**	**Bilateral** **asymmetry**	**Single** **upper limb**	**Singles** **lower limb**	**Eyeball**	**Eyelid**	**Mouth** **angle**	**Hypermotor**
Hemisphere 29 (80.6%)	1	8	22	8	0	5	4	2	0
Frontal 24 (77.4)	2	12	10	2	1	3	1	4	1
Temporal 18 (85.7%)	3	13	5	0	0	1	1	3	0
Frontotemporal 5 (100%)	1	4	1	0	0	1	2	1	0
Central 5 (100%)	0	4	5	5	0	0	1	0	0
Temporo-parieto-occipital 13 (72.%2)	1	8	3	1	0	3	4	1	0
Parieto-occipital 7 (63.6%)	0	4	1	1	0	1	3	0	0
Total: 101 (79.5%)	61		46	60					

*May be more than one ES symptom within the same video EEG recording; ES, epileptic spasm*.

The relationship among age at seizure onset and surgery, seizure type, ES semiology, EEG features, resection scope, and postoperative seizure outcomes are shown in Table 2. Statistical analysis revealed no significant differences between other factors and postoperative seizure outcomes in patients with definite focal structural lesions on MRI.

**Table 2:** The relationship between electroclinical features of ES and postoperative seizure outcome (*n* = 127).

		**Total**	**Non seizure-free**	**Seizure-free**	***P* value**
Sex (*n* %)	Male	78 (61.4)	15 (19.2)	63 (80.8)	0.66
	Female	49 (38.6)	11 (22.4)	38 (77.6)	
Age at seizure onset (months; median, 1st quartile, 3rd quartile)		7 (3,18.5)	7 (3,16)	10.75 (3.75, 24.5)	0.198
Age at seizure onset (*n* %)	≤3 yrs	117 (92.1)	24(20.5)	93 (79.5)	1.00
	>3 yrs	10 (7.9)	2 (20.0)	8 (80.0)	
Duration of epilepsy (months; median, 1st quartile, 3rd quartile)		25 (13, 42)	25 (12.85, 41.5)	28.75 (16, 48)	0.150
Age at surgery, years (*n* %)	≤3	65 (51.2)	9(16.9)	54 (83.1)	0.31
	>3	62 (48.8)	15 (24.2)	47 (75.8)	
MRI (*n* %)	MCD/LEATs	97 (76.4)	19 (19.6)	78 (80.4)	0.66
Acquired structural injury		30 (23.6)	7 (23.3)	23 (76.7)	
Seizure type (*n* %)	ES only	53 (41.7)	9 (17.0)	44 (83.0)	0.71
	ES+focal	46 (36.2)	12 (26.1)	34 (73.9)	
	ES+generalized	17 (13.4)	3 (17.6)	14 (82.4)	
	ES+generalized+focal	11 (8.7)	2 (18.2)	9 (81.8)	
ES semiology* (*n* %)	symmetric	64 (50.4)	16(25)	48 (75.0)	0.134
	asymmetric	48 (37.8)	7 (14.6)	41 (85.4)	
	Focal	79 (62.2)	24 (23.0)	55 (70.0)	
Interictal EEG (*n* %)	Generalized/Multifocal discharge	22 (17.3)	7 (31.8)	15 (68.2)	0.158
	Lateralized discharge#	25 (19.7)	7 (28.0)	18 (72.0)	
	Regional/focal discharge#	80 (63.0)	12 (15.0)	68 (85.0)	
Ictal EEG (*n* %)	Generalized/Bilateral	71 (55.9)	13 (18.3)	58 (81.7)	0.515
	lateralized	56 (44.1)	13 (23.2)	43 (76.8)	
Surgery scope (*n* %)	Focal	29 (22.8)	7 (24.1)	22 (75.9)	0.85
	Unilobar	41 (32.3)	9 (22.0)	32 (78.0)	
	Multilobar	21 (16.5)	3 (14.3)	18 (85.7)	
	Hemisphere	36 (28.3)	7 (19.4)	29 (80.6)	

*EEG, electroencephalography; ES, epileptic spasm; FCD, focal cortical dysplasia; MRI, magnetic resonance imaging; MCD, malformation of cortical development; LEATs, long-term epilepsy-associated brain tumors*.

* *May be more than one complex ES symptom within each record*.

# *May also be accompanied by generalized/multifocal discharge within each record*.

## Discussion

In recent years, great progress has been made regarding the etiology and treatment of ES. Currently, several pathogenic gene variants related to infantile spasm and other epileptic encephalopathy have been found (4, 5), which provides new insights into its pathogenesis and developing individualized treatment. Research using animal models and intracranial EEG of human ES and neuroimaging has confirmed that ES originated from focal cortical, and bilateral and focal ES were triggered through cortical-cortical and cortical-subcortical networks (6–10). Concurrently, some studies have confirmed that most cases of asymmetric ES may have a structural etiology (11). However, in this study, the cerebral structural abnormality could lead to bilateral symmetrical typical ES and/or diffusive and multifocal discharges. Nonetheless, epilepsy surgery may be still beneficial (12). Therefore, it is unreasonable to exclude surgical candidates merely according to seizure semiology and EEG. In patients with ES, the presence of focal epileptogenic lesions on MRI is the most fundamental condition for selecting surgical candidates and EZ localization. Therefore, all children with medically intractable ES caused by focal structural etiology should be considered as possible surgical candidates, regardless of the presence of focal electroclinical features (11, 13).

This retrospective study assessed 127 children with medically intractable ES and who received surgical treatment. All patients were followed up for more than 1 year, and the overall seizure-free rate was 79.5%. The seizure-free rate for 1 and 4 years was 81.1 and 72.7%, respectively. To the best of our knowledge, this is the largest single-center cohort study of this kind (12, 14–17).

In our cohort, the onset age was before 3 years in 92% of children. The etiology was either MCD or LEATs in 76.4% of patients and early acquired brain injury in 23.6% of patients. Forty-two percent of patients had only ES before surgery, while more than half had other types of seizures including focal and/or generalized onset seizures. However, the etiology and different seizure types had no significant effect on surgical outcomes. The semiological analysis of ES showed that bilateral symmetrical limb ES without signs of lateralization or axial ES had neither lateralization nor localization values. Most asymmetric ESs often showed asymmetric upper limb movement, which had a definite lateralization value with no accurate localization meaning. For example, there were multiple ocular (e.g., frontal, parietal, occipital, temporal, and central operculum) and oral motor eloquent cortex (insular-operculum network and anterior cingulate gyrus) lesions (18); thus, it is difficult to accurately localize EZ-based solely on these transient focal manifestations. Conversely, 70% of ES cases with focal lesions showed complex spasm symptoms. We speculate that different ES symptoms within the same patient should originate from the same EZ, and different ES symptoms should be caused by different brain cortex involvement in each individual seizure. In the presence of both focal onset seizures and ES, the ictal onset zone of focal seizures is more valuable for localization (19). Overall, there was no significant difference in terms of postoperative seizure-free rate between non-lateralized, asymmetric, and focal ES.

Most interictal EEGs (63%) showed constant and recurrent focal or regional epileptiform discharges, with or without generalized or multifocal discharges. This constant focal discharge was consistent with the MRI lesion location, which was of great significance for finding blurring lesions on MRI which was most likely to correspond to the EZ. Nearly 20% (*n* = 25) of patients had lateralized EEG discharges, which was valuable for EZ lateralization, but this feature lacked further significance in terms of EZ localization. Generalized or multifocal discharges without definite lateralization and localization value were found in 17.3% (*n* = 22) of patients but rarely showed typical hypsarrhythmia, possibly due to different etiologies or the age at surgery already being over infancy (20). However, there was no significant difference in the postoperative seizure-free rate among these three groups. Less than half of the patients had an ictal EEG pattern, which was of value for determining lateralization, and even less information was available in terms of EZ localization. There was no significant difference between generalized and lateralized discharges in the postoperative seizure-free rate. The EEG patterns showed that in children with ES accompanied by structural epileptogenic lesions, the interictal EEG pattern had more lateralization and localization significance than the ictal EEG pattern. As a result, generalized or multifocal discharges are not contraindications to surgery, and more attention should be paid to recognize the value of constant focal or regional discharges in the EZ lateralization and localization.

Our study showed that ES could be caused by structural lesions in multiple cortical areas. The surgical area was mainly determined using MRI, which was consistent with findings from Erdemir et al. (16). According to the MRI lesion location, the involved surgical areas were the whole hemisphere (28.3%), frontal lobe (24.4%), temporal lobe (16.5%), TPO region (14.2%), and posterior cortical region (8.7%); an isolated central region or fronto-temporo-insular region involvement was relatively rare. There was no significant difference in postoperative seizure-free rate among different lesion locations (Table 1). The poor outcomes of patients with unilateral lower limb ES (5 out of 5 patients had postoperative recurrence) may be related to incomplete resection due to the lesions being adjacent to the motor cortex, or potential EZ in other regions (21). The above results showed that ES is a more age-related than location-related epileptic phenomenon. ES with focal lesions rarely shows hypsarrhythmia, and interictal EEG often has the characteristics of focal epileptiform discharge corresponding to the epileptogenic lesions. For children with unconcordant preoperative evaluation results or unclear lesion boundaries, intracranial electrodes are helpful to find the ictal onset and early propagation of the ES (22). However, despite the paucity of intracranial evaluations, the postoperative seizure-free rate was not significantly higher in this group. Neither subdural electrodes nor SEEG can determine whether the cortical areas beyond their coverage are involved in the ES network; therefore, the final resection range is mainly based on MRI findings. Several cases underwent a second operation, primarily due to incomplete resection of the first operation and potential hemispheric lesion.

Most researchers have considered that the major factors influencing the long-term surgical outcomes of ES are seizure onset age and treatment duration of epilepsy (15); however, this study shows no such findings. Erdemir has suggested that early surgery may have positive effects on prognosis but no significant effect on long-term prognosis. Some delayed recurrence may be attributed to another EZ with time (16). Such a condition may contribute to the immature myelination of infants, which may lead to the misreading of the lesion area during neuroimaging (23).

Epileptic spasm with negative MRI findings has a variety of possible etiologies. Although mild MCD may not be ruled out, genetic, metabolic, and other causes that are contraindications for surgical treatment are more likely (24). PET with focal metabolic abnormalities and/or electroclinical manifestations is insufficient to exclude the above causes. Therefore, surgical treatment cannot be decided using PET or EEG findings alone (25). However, in very young children, it is often difficult to accurately determine the EZ location and borders. Therefore, we regarded the positivity of MRI as the basic condition for performing surgical treatment for patients with intractable ES.

In conclusion, for children with true negative MRI findings, resective surgery should be performed with great caution. When various medical treatments are ineffective, palliative treatment such as colostomy or neuromodulation should be considered. This is a single-center retrospective study, which is a study limitation; therefore, long-term follow-up should be observed in this cohort.

## Data Availability Statement

The raw data supporting the conclusions of this article will be made available by the authors, without undue reservation.

## Ethics Statement

The studies involving human participants were reviewed and approved by Institutional Review Board of the Ethics Committee of Peking University First Hospital. Written informed consent to participate in this study was provided by the participants' legal guardian/next of kin.

## Author Contributions

LC, XL, and SW designed this study and revised the manuscript. SW and CL analyzed the data and drafted and revised the manuscript. HZ collected the data. TJ, QL, YZ, and YF helped to select the patients. GY, WW, and DW helped to interpret the EEG data. HY obtained patient follow-up. All authors contributed to the article and approved the submitted version.

## Funding

This study was supported by the National Natural Science Foundation of China (grant number 82071263).

## Conflict of Interest

The authors declare that the research was conducted in the absence of any commercial or financial relationships that could be construed as a potential conflict of interest.

## Publisher's Note

All claims expressed in this article are solely those of the authors and do not necessarily represent those of their affiliated organizations, or those of the publisher, the editors and the reviewers. Any product that may be evaluated in this article, or claim that may be made by its manufacturer, is not guaranteed or endorsed by the publisher.
